# Influence of Social Determinants on Physical Performance and Geriatric Syndromes in Community-Dwelling Older Adults

**DOI:** 10.3390/ijerph22111726

**Published:** 2025-11-14

**Authors:** Roberto Israel Vázquez-Garza, Armando Martin Moreno-Amador, Carlos de la Cruz-de la Cruz, Karina Alejandra Rodriguez-Quintanilla

**Affiliations:** 1 Escuela de Medicina y Ciencias de la Salud, Tecnologico de Monterrey, Ave. Morones Prieto 3000, Monterrey 64710, NL, Mexico; israel.vazquez@tec.mx (R.I.V.-G.); armando.moreno@tec.mx (A.M.M.-A.); 2 Servicio de Hematología, Facultad de Medicina y Hospital Universitario “Dr. José Eleuterio González”, Av. Madero y Gonzalitos S/N, Monterrey 64460, NL, Mexico; cadelacruz97@gmail.com

**Keywords:** physical performance, aging, social determinants of health, geriatric syndromes, dependence

## Abstract

Background: Healthy aging involves ensuring a good quality of life and maintaining autonomy. Physical performance is a key indicator of health and autonomy in old age, and it is influenced by social determinants of health. The aim of this study was to evaluate differences in physical performance and geriatric health outcomes among older adults attending a community center, according to their educational level, pension status, and access to health services. Methods: An observational, descriptive, retrospective and cross-sectional study was carried out using the database of subjects aged 60 and over who attend a community gerontological center. Results: A total of 536 older adults (mean age 70.7 ± 8.4 years, 71.5% women, 86.9% with public health coverage) with an average age of 70.7 ± 8.4 years were included; subjects with higher education had higher weight, as well as better indicators of physical functionality: higher scores in the Barthel and Lawton–Brody indices, greater walking speed, less time in the test to get up from the chair and in the test “Time Up and Go” (TUG), and increased prehensile strength. Conclusion: Social determinants, including education, economic independence, health coverage, and pension status, significantly influence physical performance and geriatric syndromes in older adults.

## 1. Introduction

By 2050, the global population aged 60 and over is projected to double, reaching approximately 2.1 billion individuals [1]. This demographic change is particularly concerning in regions with significant socio-economic inequalities and underdeveloped health and care systems. Without targeted interventions, the societal costs—both economic and social—could be substantial, including increased healthcare expenditure and a greater burden of dependency [2]. Healthy aging is not only about living longer but about living better. It involves maintaining autonomy, functional capacity, and quality of life into old age. Physical performance is a cornerstone of healthy aging, as it reflects the integration of physiological, cognitive, and psychosocial domains that determine the ability to perform daily activities and maintain independence [3,4]. A decline in physical performance is linked to a loss of functional independence, a decrease in quality of life, a higher risk of disability, and even increased mortality [1]. Recent studies have shown that poorer physical performance, measured by slower gait speed or weaker grip strength, predicts not only mortality but also hospitalization and frailty progression in older adults across diverse settings [5]. Thus, physical performance serves as a key indicator of health and autonomy in old age, reflecting older individuals’ ability to maintain independence, prevent disability, and preserve quality of life. However, physical performance is not determined solely by biological factors or the natural aging process. It is significantly influenced by social determinants of health (SDH), which encompass the social, economic, and environmental conditions in which people are born, grow, live, work, and age [6,7]. These determinants have cumulative effects throughout life and shape health outcomes in older age. Evidence indicates that socioeconomic disadvantage—particularly lower levels of education, limited access to healthcare, and insufficient income—is associated with accelerated biological aging and poorer physical functioning [3].

Psychological and social well-being also play a protective role in maintaining functionality and delaying disability [8]. Older adults who experience strong social connections and community participation exhibit slower declines in muscle strength and gait speed [9]. Conversely, social frailty, characterized by isolation, lack of support, and low participation, has been linked to cognitive decline and reduced physical capability [10]. In low- and middle-income countries, these inequities are amplified by structural gaps in health and social protection systems. Studies in Latin American populations demonstrate that educational attainment and economic resources are crucial predictors of mobility, physical strength, and overall functionality in later life [11,12]. Furthermore, higher education and access to pensions or stable income are associated with increased physical activity and healthier lifestyle behaviors, which in turn improve physical performance [13]. These findings underscore that social context is not merely a background condition but a determinant that shapes trajectories of health and functional capacity throughout the life course. Therefore, understanding the impact of SDH on physical performance is essential to promote equitable and healthy aging. The objective of this study is to evaluate differences in physical performance and the prevalence of geriatric syndromes among older adults attending a community center, comparing groups according to their educational level, pension status, and access to health services.

## 2. Material and Methods

### 2.1. Design and Ethical Aspects

An observational, descriptive, retrospective, and cross-sectional study was carried out using the database of subjects aged 60 and over who attend a community gerontological center in Nuevo León. Each participant signed a written privacy notice authorizing the use of their sociodemographic and clinical data for epidemiological and research purposes. This authorization was obtained prospectively, in accordance with institutional and national ethical regulations for data protection and research involving human subjects. Data collection is part of a study approved by the Clinical Research Ethics Committee (CRIC) of the School of Medicine and Health Sciences of the Tecnológico de Monterrey (approval number P000879-GEROSAN-PPI-CI-CR002, 24 August 2024). To ensure confidentiality and build participants’ trust, all data were anonymized before analysis. Strict data-security measures protected participant information. A copy of the privacy notice form for authorizing use of data for research is provided as supplementary Material (Supplementary File S1).

During the preparation of this manuscript/study, the author(s) used ChatGPT5 for pur-pose of outline the introduction and organization of the text. The authors have reviewed and edited the output and take full responsibility for the content of this publication.

### 2.2. Participants

All data included in this study were selected from subjects aged 60 years and over who underwent the standardized comprehensive geriatric assessment. This assessment was routinely conducted at the community gerontology center upon admission and during follow-up. At the initial evaluation, each participant or their legal representative, when applicable, signed a written privacy notice form authorizing use of their sociodemographic and clinical data for research (Supplementary File S1). Authorization was obtained prospectively, in accordance with all ethical regulations. The data analyzed correspond to assessments performed between June 2023 and July 2024. All datasets were anonymized before analysis. No additional contact with participants was needed. Attendees comprise community-dwelling peers with diverse lifestyles, health conditions, and socioeconomic backgrounds.

The sampling frame included data of all older adults registered in the gerontological center’s database during the specified study period who underwent a complete geriatric evaluation. We included data from individuals aged ≥60 years who attended the center during this period and had complete documentation for the variables of interest in their clinical records. Incomplete, duplicated, or missed records were excluded from the analysis. A non-probabilistic consecutive sampling method was used, ensuring that every available case meeting the inclusion criteria was incorporated. Accordingly, there was no formal sample size calculation because the total eligible population with complete data during the study period comprised the study population. All participants with eligible data were included, and as data were obtained solely from existing clinical files, there were no refusals or additional dropouts after eligibility was established. This ensured a comprehensive and transparent participant flow from registration, through assessment, to data inclusion.

### 2.3. Measurements

The analyzed data correspond to those obtained during the initial geriatric assessment of subjects entering the community gerontological center. These data include sociodemographic information, comorbidities, medication lists, and the degree of functional dependence as measured by the Barthel Index, as well as the results of various physical performance tests: walking speed, hand grip strength, and the Timed Up and Go (TUG) test. These tests were selected for their predictive value for disability and autonomy in older adults. Walking speed, grip strength, and TUG are well-established indicators of physical performance, which directly relate to functional independence and the ability to perform daily activities. This links them to the core theme of autonomy in aging. In addition to these core variables, data collection included anthropometric, functional, clinical, and geriatric parameters, obtained through standardized evaluation. Anthropometric measurements included body weight (kg) and body mass index (BMI, kg/m^2^). Functional dependence was assessed with the Barthel Index (points), which measures performance in ten basic activities of daily living. Scores range from 0 to 100; higher values indicate greater independence. A score of 60 or less reflected moderate-to-severe dependence. Instrumental functionality was evaluated using the Lawton–Brody Index, which assesses the ability to perform complex daily tasks, such as managing finances, medications, and household activities. Higher scores on this index reflect greater autonomy.

Physical performance was evaluated using standardized protocols. Gait speed was measured over a 4-m distance at the participant’s usual walking pace using a digital stopwatch. Results were expressed in meters per second (m/s). The chair stand test quantified lower-limb strength and endurance by recording the time, in seconds (n = 490), required to rise from a seated position five times without using the arms. The TUG test measured mobility and balance by timing, in seconds, the period needed to stand up from a chair, walk three meters, turn, return, and sit down again. Hand grip strength of the dominant hand was measured in kilograms using a calibrated Jamar dynamometer. Each participant completed three trials; the maximum value was recorded.

Comorbidity burden was quantified using the Charlson Comorbidity Index (CCI), and the number of medications and supplements was recorded to assess polypharmacy, defined as the regular use of five or more drugs. Fall risk was classified as low, moderate, or high according to standardized geriatric scales, and vaccination status was recorded as complete, incomplete, or none, based on immunization documentation. Furthermore, the presence of geriatric syndromes was documented as part of the comprehensive geriatric assessment. The syndromes included in this study were neurocognitive impairment, weight loss, hyporexia, sensory impairment, and depression. These were identified based on the information in medical records and standardized screening tools used during the assessment. For the purposes of this study, “Geriatric Syndromes” were operationally defined as the presence of one or more of these conditions, representing multidimensional vulnerability and cumulative physiological decline associated with aging.

Vaccination status was included in the geriatric assessment and classified into three categories: none, incomplete, and complete. A “complete” vaccination scheme followed the national adult immunization schedule of the Mexican Ministry of Health for people 60 and over. This includes vaccines for: seasonal influenza (annual), pneumococcal (either PPSV23 or PCV13, per national guidelines), tetanus-diphtheria (Td) or tetanus-diphtheria-pertussis (Tdap) booster, and COVID-19 as recommended. Participants with all these vaccines were classified as complete. The incomplete status referred to people who received at least one but not all recommended vaccines. “None” indicated no documented vaccines. To minimize recall bias and ensure data reliability, vaccination information was obtained from official records rather than self-reports. The vaccination card or certificate of every participant was reviewed, as these documents are required in medical files at the community gerontological center. Only verified data were analyzed.

Educational level was classified into six categories according to years of formal education: none (0 years), primary (1–6 years), secondary (7–9 years), high school (10–12 years), bachelor’s degree (>12 years), and postgraduate studies. Pension status was recorded as “yes” for participants receiving any regular government or private retirement benefit, and “no” for those without pension income. Health coverage was categorized as public (IMSS, ISSSTE, or INSABI), mixed (public and private), or none.

### 2.4. Analysis

A descriptive analysis of the sociodemographic, anthropometric, physical performance, and comorbidities was carried out. Quantitative variables were expressed as mean, standard deviation, or median and interquartile range, depending on distribution evaluated by the Kolmogorov–Smirnov test. Qualitative variables were presented as absolute frequencies and percentages. For descriptive variables, 95% confidence intervals (CIs) were calculated using the Wilson method to give precision estimates for proportions reported. For group comparisons (by gender, educational level, type of medical security, economic dependence, and pension receipt), Student’s *t*-test or Mann–Whitney U test was used for quantitative variables. The χ^2^ or Fisher exact test was used for qualitative variables. For bivariate analyses, 95% CIs were not calculated because these results corresponded to raw data from the population. All statistical tests were two-tailed. A *p*-value < 0.05 was considered statistically significant. Missing data were handled through case-wise deletion, and only participants with complete information for variables of interest were included in the final analyses to preserve data integrity and reduce bias. Analysis was performed with the IBM SPSS Statistics package, version 27.

## 3. Results

From a total of 555 older adults, data from 536 subjects were analyzed to assess their social determinants of health. The mean age was 70.7 ± 8.4 years, and 71.5% were women. Most participants (86.9%) had public health insurance. Nearly half were married (47.4%), and 28.9% held a bachelor’s degree. Financial independence was reported by 63.8% of participants. Additionally, 87.5% received a pension, and 48.9% had financial support from family members. The demographic and social determinants of health are summarized in Table 1.

The most prevalent geriatric syndromes (n = 526) were sensory impairment, present in 85.4% (95% CI 82.1–88.2) of older adults, followed by neurocognitive impairment, identified in 48.9% (95% CI 44.7–53.2) of cases, and depression, with a frequency of 46.0% (95% CI 41.8–50.3). Less frequently, hyporexia was observed in 19.4% (95% CI 16.2–23.0) of participants and weight loss in 16.7% (95% CI 13.8–20.1).

### Analysis of Social Determinants

In the subsequent analyses, variables related to physical performance, comorbidities, vaccination history, and geriatric syndromes included only those patients for whom complete classification data were available.

In the gender-based comparison, men exhibited higher weight, dominant handgrip strength, and gait speed, whereas women had a significantly higher body mass index (BMI). Differences were also observed in the Barthel score, with slightly higher values among men. No significant differences were found in fall risk, polypharmacy, or most comorbidity indicators. Regarding geriatric syndromes, men had a higher prevalence of neurocognitive impairment (56.7% vs. 45.7%), while women showed more cases of hyporexia (21.5% vs. 14%) and depression (49.2% vs. 38%) (Table 2).

Regarding educational level, older adults with higher education presented greater weight, as well as better indicators of physical functionality: higher scores on the Barthel and Lawton–Brody indices, greater gait speed, shorter times on the chair stand test and the “Time Up and Go” (TUG) test, and greater handgrip strength. They also showed lower risk of falls and a lower Charlson comorbidity index. In relation to geriatric syndromes, those with only basic education had higher prevalence of neurocognitive impairment (57% vs. 33.5%), weight loss (19.8% vs. 11%), hyporexia (22.7% vs. 13.2%), and depression (51.7% vs. 35.2%), suggesting an association between lower educational level and increased vulnerability to these syndromes (Table 3).

In the analysis by type of medical insurance, differences were identified in Barthel scores, with lower values among those without medical coverage. The rest of the anthropometric variables, physical functionality, and vaccination history did not show statistically significant differences. When analyzing geriatric syndromes, weight loss was significantly more frequent among those with private or mixed insurance (33.3%), whereas no relevant differences were observed in the other conditions evaluated (neurocognitive impairment, hyporexia, sensory impairment, and depression), according to insurance coverage (Table 4).

Regarding the degree of economic dependence, significant differences were found in Barthel and Lawton–Brody scores, gait speed, TUG, grip strength, and the number of medications used. Older adults with economic independence showed better physical performance indicators and a lower prevalence of polypharmacy. With respect to geriatric syndromes, no statistically significant differences were observed; however, there was a trend toward higher depression among those with complete dependence (55% vs. 42.7% in independent individuals) and higher hyporexia among those with partial dependence (26.1%) (Table 5).

Finally, regarding pension reception, those who did not receive a pension showed better gait speed, shorter times on the chair stand test and TUG, as well as lower Charlson comorbidity index and fewer medications consumed. Polypharmacy was more frequent among those receiving a pension. Concerning geriatric syndromes, sensory impairment was more common among pension recipients (86.8% vs. 75.4%), while the remaining conditions (neurocognitive impairment, weight loss, hyporexia, and depression) did not show significant differences (Table 6).

## 4. Discussion

The findings of this study show associations, not causal relationships. There are clear disparities in physical performance and geriatric syndromes by social factors such as gender, education, and socio-economic status. The cross-sectional design prevents causal inference and suggests possible reverse causality. For example, poor physical performance could lower access to employment or pension benefits, rather than the opposite.

First, men showed better physical performance than women. They had higher grip strength, walking speed, and more autonomy in daily activities, as measured by the Barthel index. This matches the literature. Although women live longer, they tend to have more limitations and spend more years with disability [14,15]. Older women, even when age and comorbidities are considered, report more difficulties with mobility and daily activities than men [16]. Biological factors play a role. Women often have lower muscle mass and more chronic diseases after a longer life. However, social and structural factors also have a major impact. In Latin America, older women often had less access to education, paid work, and physical activity. Across a lifetime, these disadvantages reduce functional reserve in old age [17]. Gender is viewed as a risk factor for worse physical function, shaped by role and opportunity inequalities. Promoting gender equity could help close this gap [16]. In short, our findings reflect long-term inequities. Women experience greater functional challenges despite their longer lifespan. This highlights the need for fair interventions that promote physical activity, social participation, and access to preventive care for everyone.

Second, education stood out as a key determinant. Older people with more education had better physical functionality and fewer geriatric syndromes, including less cognitive impairment and depression. This matches recent evidence. Formal education, as a social determinant, positively affects cognitive and functional aging [6]. Higher education gives better economic opportunities, stronger health literacy, and healthier lifestyles. Together, these factors build cognitive and physiological reserves, which help resist age decline [17]. Studies in Latin America find older adults with higher education have less risk of cognitive impairment [18]. The socio-economic level linked to education also results in better physical condition. Research shows those with higher education and income are less limited in daily activities and perform better in physical tests. This helps to explain the differences between genders seen above [16]. In our study, lower levels of education may be linked to more frequent depression, cognitive impairment, and other syndromes. Education works as lifelong protection—improving coping skills, information access, and support networks. These findings highlight the role of education and lifelong learning in healthy aging. It gives older people tools to maintain function and independence [6].

On economic factors, economic independence is linked to improved physical performance in the elderly we studied. Older adults with enough income or financial autonomy had more strength and mobility. This fits with evidence: higher socioeconomic status protects against frailty and disability in old age [19]. One study found that older adults with higher incomes are about half as likely to be frail as lower-income peers [19]. Economic security brings better nutrition, housing, and healthcare, and reduces stress from poverty. However, pension receipts had mixed results in our study. Not all pension recipients had better health, implying a more complex impact. Those with only minimum, non-contributory pensions may still face insecurity. Some without formal pensions may receive family support or other income. The literature echoes this complexity. Non-contributory pensions in Latin America reduce old-age poverty and increase medical service use. For example, in Mexico, older pensioners use medical care more often and have more public insurance [20]. Pension scheme design matters. In Yucatán, monthly pensions (vs bimonthly) led to fewer limitations and less need for care [21]. This suggests that regular, sufficient pensions help maintain autonomy [16]. Yet, those with better function may also qualify for income-generating activities or benefits, indicating possible reverse causality. Some studies do not show significant health improvement from pensions, beyond more service use; this matches our data [15]. The benefit may depend on pension amount, frequency, and each recipient’s circumstances. For vulnerable groups, pensions may help, but not erase, lifelong inequalities. Our findings suggest that more research is needed on how social protection affects older adults’ health in Mexico.

An interesting finding in our study was the higher frequency of weight loss among participants with private or mixed health insurance. Although this result may seem counterintuitive, it can be interpreted within the context of healthcare access and socioeconomic status. Individuals with private or dual coverage generally have greater contact with medical services, leading to earlier recognition and documentation of unintentional weight loss—a reflection of diagnostic vigilance rather than higher prevalence. Similar studies have reported that populations with better health coverage are more frequently screened for and diagnosed with age-related or chronic conditions, including those associated with involuntary weight loss [20,21].

Part of this group may also engage in intentional weight reduction after medical advice for obesity or metabolic disorders. These conditions are more actively managed in private healthcare. So, the observed association may show differences in health-seeking behavior, chronic disease management, or intentional versus unintentional weight changes, not a true increase in pathological weight loss. These findings highlight how social and structural factors, especially healthcare access, affect the occurrence and detection of health conditions in older adults.

Together, these results underline the role of social determinants in creating health gaps in older adults. The advantages seen in men, those with higher education, and those with better economic status reflect structural inequities in society. From an equity view, it is concerning that traditionally disadvantaged groups—women, the less educated, and people with limited income—face a greater burden of physical decline and geriatric syndromes. Regional evidence also suggests a link between social inequalities and poorer health in older adults. In Latin America, high gender and socio-economic inequalities result in more dementia and earlier age-related illnesses, especially in women [17]. Recent multinational studies suggest that, in Latin American countries, factors such as education, income, and social support influence healthy aging even more than age or sex. This reveals different patterns linked to development and equity levels in each context [6]. Thus, these differences are not only biomedical but also reflect the accumulation of social injustice. Potential mechanisms explaining these findings include differential access to material resources, healthcare, social participation, and opportunities for physical activity, as well as differences in cognitive and psychosocial reserve across the lifespan. To achieve equitable healthy aging, public health policies must reduce these gaps. This means ensuring both genders have equal opportunities to maintain their function, supporting older adults with limited education through preventive care and health education, and providing proper social protection for the most vulnerable, including decent pensions and universal health coverage. Enabling people to age healthily in Latin America requires addressing the social determinants of health and promoting environments where older adults can fulfill their potential and participate in society, regardless of gender, education, or economic status [22].

These results should be interpreted considering the sample’s sociocultural context. Drawn from a single urban gerontological center in northern Mexico, the findings may not represent rural or more diverse populations, affecting external validity. However, they offer relevant implications for policies promoting functional independence in older adults within this context. It is important to note the methodological limitations of this study. The cross-sectional design is appropriate for estimating associations within a community-dwelling older adult population. It is supported by a reasonable sample size (n = 536). However, this design prevents the establishment of direct causal relationships and is vulnerable to reverse causality. Exposure and outcome variables were measured simultaneously. The associations we found cannot determine direction or rule out confounding factors. We only provide a “snapshot” of health at a single point in time. We cannot assess changes over time. To minimize potential selection bias, the study included all individuals aged 60 years or older who attended the community gerontological center during the study period and had complete data for all variables of interest in their clinical records were included. A non-probabilistic consecutive sampling method was used, incorporating all eligible cases to maximize representativeness and reduce systematic exclusion.

Additionally, participants were recruited from a community gerontological center in Nuevo León using convenience sampling, specifically by including all attendees who met eligibility criteria during the study window. This recruitment strategy, based on ease of access rather than random selection from the wider population, may limit the generalizability of our findings to the broader elderly population. People attending a community center may have different characteristics compared to those who do not. The urban setting of the center may not capture the health realities in other regions of the country or Latin America, such as rural or socioeconomically diverse locations.

Another limitation is the potential for survival bias. Our cohort includes only older adults who survived and are healthy or functional enough to join the center and participate. Thus, frail or severely ill individuals may have died earlier or not attended. As a result, we could underestimate the prevalence of severe geriatric syndromes. Another possible bias relates to self-care and participation. Attending a gerontological center may mean some participants have better health habits, such as staying socially and physically active, compared to others of their age. This selection bias could make us estimate more conservative associations. For example, educational or economic differences might be greater in the general population if those who are less educated or poorer—and do not attend—have even worse health outcomes.

Finally, several variables, such as geriatric syndromes, rely on screening or self-report. This may cause underreporting (for example, undiagnosed depression) or reporting bias. However, the study attempted to minimize recall and classification biases by confirming information with clinical records and standardized geriatric assessments recorded in each older adult’s medical file. Residual confounding from unmeasured factors such as nutritional status, depressive symptoms, or lifestyle habits cannot be excluded, and future longitudinal studies should explore these relationships in more depth.

## 5. Conclusions

This observational study conducted at a gerontological center in Mexico revealed significant associations, rather than causal effects, between social factors—including gender, education, and socioeconomic status—and healthy aging. Older men demonstrated better physical performance than women. Individuals with higher levels of education and financial independence exhibited higher functionality and a lower burden of geriatric syndromes. However, the effect of receiving a pension was not uniform. This suggests that current social protection may be insufficient or uneven in its health benefits.

These findings should be interpreted within the social and economic context of the studied population. The mechanisms linking education and income to functional health may operate through several pathways. These include access to resources, healthcare, cognitive reserve, and opportunities for physical activity. Taken together, these results highlight the need to strengthen protective social factors in old age. It is essential to promote environments that ensure gender equity, access to education, and continuous participation. Economic security through adequate pensions and support and accessibility to quality health services throughout the life course are also key for active aging.

Only through comprehensive strategies that combine social and health interventions can we close the observed gaps. These strategies can help ensure that increased longevity is accompanied by healthier and fuller lives. Ultimately, this study provides evidence to promote actions that support aging with dignity and well-being. We call on researchers and decision-makers to conduct longitudinal studies that explore the social determinants and their influence on health more deeply. Such efforts will help clarify directionality and assess causality. They will also guide realistic policy actions within the Latin American sociocultural context. This can foster equitable and healthy aging across the region.

## Figures and Tables

**Table 1:** Demographic characteristics and social determinants of subjects.

Variables	n	Proportion (95% CI)
Gender		
Female	383	71.5 (67.5–75.1)
Male	153	28.5 (24.9–32.5)
Medical security		
Public	466	86.9 (83.8–89.5)
Private ± Public	33	6.2 (4.4–8.5)
None	37	6.9 (5.0–9.4)
Civil status		
Married	254	47.4 (43.2–51.6)
Divorced or separated	67	12.5 (10.0–15.6)
Single	38	7.1 (5.2–9.6)
Widowed	172	32.1 (28.3–36.2)
Other	5	0.9 (0.4–2.2)
Educational level		
None (0 years)	4	0.7 (0.3–1.9)
Primary (1–6 years)	126	23.5 (20.1–27.3)
Secondary Education (7–9 years)	107	20.0 (16.8–23.6)
High school (10–12 years)	113	21.1 (17.8–24.7)
Bachelor’s degree	155	28.9 (25.2–32.9)
Postgraduate	31	5.8 (4.1–8.1)
Economic dependency		
Complete dependency	82	15.3 (12.5–18.6)
Partial dependency	112	20.9 (17.7–24.5)
Economic independency	342	63.8 (59.7–67.8)
Receives financial pension	469	87.5 (84.4–90.0)
Financial support from family	262	48.9 (44.7–53.1)

Data are expressed as n and % (95% confidence interval, 95% CI), as appropriate.

**Table 2:** Association of gender with anthropometry, physical performance, comorbidities, vaccination status, and geriatric syndromes in patients.

Variable	Women	Men	*p*
Anthropometric variables			
Weight (kg)	70.3 ± 14.3	77.8 ± 16.5	<0.001
BMI (kg/m^2^)	29.2 ± 5.6	27.6 ± 5.4	0.002
Functional performance			
Barthel (points, n = 527)	100 (95–100)	100 (100–100)	0.008
Lawton–Brody (points, n = 527)	8 (8–8)	8 (7–8)	0.052
Gait speed (m/s, n = 498)	1.02 (0.82–1.20)	1.16 (0.89–1.36)	<0.001
Chair stand test (seconds, n = 490)	12.7 (10.6–15.2)	12.6 (10.3–15.9)	0.884
TUG (seconds, n = 500)	8.9 (7.8–10.9)	8.6 (7.2–11.3)	0.258
Dominant handgrip strength (kg, n = 509)	17.5 (14.0–21.0)	25.9 (21.7–31.5)	<0.001
Comorbidities and medication use			
CCI (points)	1 (0–2)	1 (0–2)	0.52
Number of drugs and/or supplements	4 (1–6)	4 (1–5)	0.366
Polypharmacy	204 (53.3%)	79 (51.6%)	0.733
Fall risk (n = 475)	-		0.848
Low risk	256 (74.6%)	99 (75.0%)	
Moderate risk	20 (5.8%)	6 (4.5%)	
High risk	67 (19.5%)	27 (20.5%)	
Vaccination status			0.191
None	23 (6.0%)	6 (3.9%)	
Incomplete	296 (77.3%)	129 (84.3%)	
Complete	64 (16.7%)	18 (11.8%)	
Geriatric syndromes (n = 526)			
Neurocognitive impairment	172 (45.7%)	85 (56.7%)	0.024
Weight loss	66 (17.6%)	22 (14.7%)	0.423
Hyporexia	81 (21.5%)	21 (14.0%)	0.048
Sensory impairment	315 (83.8%)	134 (89.3%)	0.104
Depression	185 (49.2%)	57 (38.0%)	0.02

Data presented as mean ± SD, median (Q1–Q3), or n (%), as appropriate. Statistical tests used: Student’s *t*-test or Mann–Whitney U test for quantitative variables; Chi-square or Fisher’s exact test for categorical variables. BMI: body mass index; TUG: Timed Up and Go; CCI: Charlson Comorbidity Index.

**Table 3:** Association of educational level with anthropometry, physical performance, comorbidities, vaccination status, and geriatric syndromes in patients.

Variable	Higher Education (>12 Years)	Basic Education (≤12 Years)	*p*
Anthropometric variables			
Weight (kg)	75.8 ± 16.4	70.6 ± 14.4	<0.001
BMI (kg/m^2^)	29.1 ± 5.8	28.6 ± 5.4	0.339
Functional performance			
Barthel (points, n = 527)	100 (100–100)	100 (95–100)	<0.001
Lawton–Brody (points, n = 527)	8 (8–8)	8 (7–8)	<0.001
Gait speed (m/s, n = 498)	1.16 (0.97–1.36)	0.98 (0.79–1.18)	<0.001
Chair stand test (seconds, n = 490)	11.4 (9.9–13.7)	13.5 (11.5–16)	<0.001
TUG (seconds, n = 500)	8 (7–9.5)	9.4 (8–11.9)	<0.001
Dominant handgrip strength (kg, n = 509)	22 (17.6–26.2)	18.1 (14.3–23.0)	<0.001
Comorbidities and medication use			
CCI (points)	1 (0–2)	2 (0–2)	0.034
Number of drugs and/or supplements	3 (1–6)	4 (1–6)	0.982
Polypharmacy	93 (50.0%)	190 (54.3%)	0.344
Fall risk (n = 475)	-		<0.001
Low risk	136 (82.9%)	219 (70.4%)	
Moderate risk	11 (6.7%)	15 (4.8%)	
High risk	17 (10.4%)	77 (24.8%)	
Vaccination status			0.402
None	8 (4.3%)	21 (6.0%)	
Incomplete	145 (78.0%)	280 (80.0%)	
Complete	33 (17.7%)	49 (14.0%)	
Geriatric syndromes (n = 526)			
Neurocognitive impairment	61 (33.5%)	196 (57.0%)	<0.001
Weight loss	20 (11.0%)	68 (19.8%)	0.010
Hyporexia	24 (13.2%)	78 (22.7%)	0.009
Sensory impairment	156 (85.7%)	293 (85.2%)	0.868
Depression	64 (35.2%)	178 (51.7%)	<0.001

Data presented as mean ± SD, median (Q1–Q3), or n (%), as appropriate. Statistical tests used: Student’s *t*-test or Mann–Whitney U test for quantitative variables; Chi-square or Fisher’s exact test for categorical variables. BMI: body mass index; TUG: Timed Up and Go; CCI: Charlson Comorbidity Index.

**Table 4:** Association of type of medical insurance with anthropometry, physical performance, comorbidities, vaccination status, and geriatric syndromes in patients.

Variable	Public	Private ± Public	No Insurance	*p*
Anthropometric variables				
Weight (kg)	72.6 ± 15.4	72.2 ± 13.5	71 ± 16.1	0.845
BMI (kg/m^2^)	28.8 ± 5.6	29.2 ± 5.0	28.3 ± 5.7	0.788
Functional performance				
Barthel (points, n = 527)	100 (95–100)	100 (95–100)	97 (90–100)	0.037
Lawton–Brody (points, n = 527)	8 (7–8)	8 (6–8)	8 (5–8)	0.229
Gait speed (m/s, n = 498)	1.04 (0.84–1.25)	1.19 (0.90–1.37)	1.06 (0.84–1.18)	0.243
Chair stand test (seconds, n = 490)	12.8 (10.6–15.5)	11.6 (10.7–13.7)	12.6 (10.2–15.5)	0.158
TUG (seconds, n = 500)	8.9 (7.7–11.0)	8.2 (6.7–9.7)	8.5 (7.2–10.0)	0.081
Dominant handgrip strength (kg, n = 509)	19.7 (15.1–24.4)	20.1 (16.5–24.8)	17.1 (13.5–23.8)	0.206
Comorbidities and medication use				
CCI (points)	1 (0–2)	1 (0–2)	1 (0–1)	0.606
Number of drugs and/or supplements	4 (1–6)	5 (1–7)	3 (1–4)	0.185
Polypharmacy	247 (53.0%)	20 (60.6%)	16 (43.2%)	0.338
Fall risk (n = 475)	-			0.872
Low risk	309 (75.2%)	22 (75.9%)	24 (68.6%)	
Moderate risk	22 (5.4%)	1 (3.4%)	3 (8.6%)	
High risk	80 (19.5%)	6 (20.7%)	8 (22.9%)	
Vaccination status				0.555
None	23 (4.9%)	3 (9.1%)	3 (8.1%)	
Incomplete	269 (79.2%)	25 (75.8%)	31 (83.8%)	
Complete	74 (15.9%)	5 (15.2%)	3 (8.1%)	
Geriatric syndromes (n = 526)				
Neurocognitive impairment	230 (50.1%)	12 (36.4%)	15 (44.1%)	0.265
Weight loss	73 (15.9%)	11 (33.3%)	4 (11.8%)	0.025
Hyporexia	89 (19.4%)	7 (21.2%)	6 (17.6%)	0.934
Sensory impairment	396 (86.3%)	26 (78.8%)	27 (79.4%)	0.3
Depression	215 (46.8%)	10 (30.3%)	17 (50.0%)	0.163

Data presented as mean ± SD, median (Q1–Q3), or n (%), as appropriate. Statistical tests used: Student’s *t*-test or Mann–Whitney U test for quantitative variables; Chi-square or Fisher’s exact test for categorical variables. BMI: body mass index; TUG: Timed Up and Go; CCI: Charlson Comorbidity Index.

**Table 5:** Association of economic dependency with anthropometry, physical performance, comorbidities, vaccination status, and geriatric syndromes in patients.

Variable	Complete	Partial	No Economic Dependency	*p*
Anthropometric variables				
Weight (kg)	72.6 ± 15.4	72.2 ± 13.5	71.0 ± 16.1	0.453
BMI (kg/m^2^)	28.8 ± 5.6	29.2 ± 5	28.3 ± 5.7	0.09
Functional performance				
Barthel (points, n = 527)	100 (95–100)	100 (95–100)	97 (90–100)	0.003
Lawton–Brody (points, n = 527)	8 (7–8)	8 (6–8)	8 (5–8)	0.003
Gait speed (m/s, n = 498)	1.04 (0.84–1.25)	1.19 (0.9–1.37)	1.06 (0.84–1.18)	0.001
Chair stand test (seconds, n = 490)	12.8 (10.6–15.5)	11.6 (10.7–13.7)	12.6 (10.2–15.5)	0.572
TUG (seconds, n = 500)	8.9 (7.7–11)	8.2 (6.7–9.8)	8.5 (7.2–10)	0.017
Dominant handgrip strength (kg, n = 509)	19.7 (15.1–24.4)	20.1 (16.5–24.8)	17.1 (13.5–23.8)	<0.001
Comorbidities and medication use				
CCI (points)	1 (0–2)	1 (0–2)	1 (0–1)	0.122
Number of drugs and/or supplements	4 (1–6)	5 (1–7)	3 (1–4)	0.007
Polypharmacy	32 (39.0%)	62 (55.4%)	189 (55.3%)	0.025
Fall risk (n = 475)	-			0.051
Low risk	46 (66.7%)	69 (69.7%)	240 (78.2%)	
Moderate risk	5 (7.2%)	10 (10.1%)	11 (3.6%)	
High risk	18 (26.1%)	20 (20.2%)	56 (18.2%)	
Vaccination status				0.692
None	4 (4.9%)	4 (3.6%)	21 (6.1%)	
Incomplete	68 (82.9%)	88 (78.6%)	269 (78.7%)	
Complete	10 (12.2%)	20 (17.9%)	52 (15.2%)	
Geriatric syndromes (n = 526)				
Neurocognitive impairment	44 (55.0%)	60 (54.1%)	153 (45.7%)	0.152
Weight loss	10 (12.5%)	24 (21.6%)	54 (16.1%)	0.221
Hyporexia	16 (20.0%)	29 (26.1%)	57 (17.0%)	0.108
Sensory impairment	64 (80.0%)	97 (87.4%)	288 (86.0%)	0.316
Depression	44 (55.0%)	55 (49.5%)	143 (42.7%)	0.098

Data presented as mean ± SD, median (Q1–Q3), or n (%), as appropriate. Statistical tests used: Student’s *t*-test or Mann–Whitney U test for quantitative variables; Chi-square or Fisher’s exact test for categorical variables. BMI: body mass index; TUG: Timed Up and Go; CCI: Charlson Comorbidity Index.

**Table 6:** Association between pension reception and anthropometry, physical performance, comorbidities, vaccination status, and geriatric syndromes in patients.

Variable	Pension Received	Pension Not Received	*p*
Anthropometric variables			
Weight (kg)	72.6 ± 15.4	71.4 ± 15.0	0.568
BMI (kg/m^2^)	28.8 ± 5.5	28.5 ± 5.7	0.687
Functional performance			
Barthel (points, n = 527)	100 (95–100)	100 (98–100)	0.09
Lawton–Brody (points, n = 527)	8 (7–8)	8 (8–8)	0.05
Gait speed (m/s, n = 498)	1.03 (0.83–1.25)	1.11 (0.96–1.30)	0.024
Chair stand test (seconds, n = 490)	13 (10.9–15.6)	11.1 (9.5–12.8)	<0.001
TUG (seconds, n = 500)	9 (7.7–11.2)	8 (6.8–9.0)	<0.001
Dominant handgrip strength (kg, n = 509)	19.3 (14.9–24.4)	20.8 (16.7–23.8)	0.226
Comorbidities and medication use			
CCI (points)	1 (0–2)	0 (0–1)	0.001
Number of drugs and/or supplements	4 (1–6)	2 (0–5)	0.004
Polypharmacy	257 (54.8%)	26 (38.8%)	0.014
Fall risk (n = 475)	-		0.701
Low risk	306 (74.1%)	49 (79.0%)	
Moderate risk	23 (5.6%)	3 (4.8%)	
High risk	84 (20.3%)	10 (16.1%)	
Vaccination status			0.83
None	26 (5.5%)	3 (4.5%)	
Incomplete	370 (78.9%)	55 (82.1%)	
Complete	73 (15.6%)	9 (13.4%)	
Geriatric syndromes (n = 526)			
Neurocognitive impairment	227 (49.2%)	30 (46.2%)	0.641
Weight loss	79 (17.1%)	9 (13.8%)	0.506
Hyporexia	92 (20.0%)	10 (15.4%)	0.383
Sensory impairment	400 (86.8%)	49 (75.4%)	0.015
Depression	215 (46.6%)	27 (41.5%)	0.44

Data presented as mean ± SD, median (Q1–Q3), or n (%), as appropriate. Statistical tests used: Student’s *t*-test or Mann–Whitney U test for quantitative variables; Chi-square or Fisher’s exact test for categorical variables. BMI: body mass index; TUG: Timed Up and Go; CCI: Charlson Comorbidity Index.

## Data Availability

The data used in this study are available from the corresponding author upon reasonable request.

## Supplementary Materials

The following supporting information can be downloaded at: https://www.mdpi.com/article/10.3390/ijerph22111726/s1, File S1: Integral Customer Privacy Notice.
