# Supplementary material for: Influence of Social Determinants on Physical Performance and Geriatric Syndromes in Community-Dwelling Older Adults
Source: Int J Environ Res Public Health. 2025 Nov 14;22(11):1726. doi: 10.3390/ijerph22111726 (PMC12652700; doi:10.3390/ijerph22111726)
Supplement: Supplementary file 1 [file ijerph-22-01726-s001.zip › ijerph-3874837-supplementary.pdf]

Aviso de Privacidad Clientes Integral

Unidad Gerontológica San Nicolás (nombre comercial: UNIDAD GERONTOLÓGICA SAN NICOLAS I CENTRO GERONTOLÓGICO SAN NICOLAS) con domicilio en Av. Lerdo de Tejada 412B, Sin Nombre de Col 5, 66428 San Nicolás de los Garza, N.L., es el responsable del uso y protección de sus datos personales, y al respecto le informamos lo siguiente:

Departamento de Privacidad

Para atender cualquier solicitud acerca del ejercicio de sus derechos ARCO, para la revocación del consentimiento que nos haya otorgado del tratamiento de sus datos personales, para limitar el uso o divulgación de sus datos, o acerca de las finalidades para el tratamiento de sus datos personales, ponemos a su disposición nuestro Departamento de Privacidad en los siguientes medios de contacto:

Correo electrónico: **vidaplenagerontologico@gmail.com**

Teléfono: **81 1967 2260**

Para poder comunicarnos con usted, necesitamos los siguientes datos personales y de contacto en su solicitud:

- **Nombre del titular**
- **Carta de autorización en caso de que la solicitud la haga otra persona que no sea el titular**
- **Copia de identificación del titular**
- **Correo electrónico**
- **Teléfono**

Después de recibir su solicitud, recibirá nuestra respuesta en un plazo máximo de veinte días hábiles por los medios de contacto que nos proporcione.

¿Para qué fines utilizaremos sus datos personales?

Los datos personales que recabamos de usted, los utilizaremos para las siguientes finalidades que son necesarias para el servicio que solicita:

- Para fines de contacto
- Para facturación y cobro
- Para elaborar un expediente clínico
- Para estudios observacionales y epidemiológicos

De manera adicional, utilizaremos su información personal para las siguientes finalidades secundarias que no son necesarias para el servicio solicitado, pero que nos permiten y facilitan brindarle mejor atención:

- Para envío de publicidad de nuestros eventos

En caso de que no desee que sus datos personales sean tratados para estos fines adicionales, lo podrá indicar en el medio a través del cual nos proporcione sus datos personales, seleccionando la opción correspondiente.

La negativa para el uso de sus datos personales para estas finalidades adicionales no podrá ser un motivo para que le neguemos los servicios y productos que solicita o contrata con nosotros.

¿Qué datos personales utilizaremos para estos fines?

Para llevar a cabo las finalidades descritas en el presente Aviso de Privacidad, utilizaremos los siguientes datos personales:

- Datos de contacto
- Datos de identificación
- Datos de facturación
- Datos laborales
- Datos académicos

Además de los datos personales mencionados anteriormente, para las finalidades informadas en el presente Aviso de Privacidad utilizaremos los siguientes datos personales considerados como sensibles, que requieren de especial protección:

- Datos de salud
  - Peso, talla, fuerza de prensión, circunferencia de pantorrilla, velocidad de la marcha
  - Información obtenida durante su evaluación geriátrica integral tales como: escolaridad, edad, estado civil, funcionalidad, nivel socio-económico, nivel

cognitivo y estado de ánimo, número de medicamentos utilizados, diagnósticos médicos presentes.

¿Cómo puede Acceder, Rectificar o Cancelar sus datos personales, u Oponerse a su uso?

Usted tiene derecho a conocer qué datos personales tenemos de usted, para qué los utilizamos y las condiciones del uso que les damos (Acceso). Asimismo, es su derecho solicitar la corrección de su información personal en caso de que esté desactualizada, sea inexacta o incompleta (Rectificación); que la eliminemos de nuestros registros o bases de datos cuando considere que la misma no está siendo utilizada conforme a los principios, deberes y obligaciones previstas en la normativa (Cancelación); así como oponerse al uso de sus datos personales para fines específicos (Oposición). Estos derechos se conocen como derechos ARCO.

Si usted desea ejercer sus derechos ARCO, lo podrá hacer contactándose con nuestro Departamento de Privacidad en los medios establecidos en el presente aviso.

¿Cómo puede revocar su consentimiento para el uso de sus datos personales?

Usted puede revocar el consentimiento que, en su caso, nos haya otorgado para el tratamiento de sus datos personales. Sin embargo, es importante que tenga en cuenta que no en todos los casos podremos atender su solicitud o concluir el uso de forma inmediata, ya que es posible que por alguna obligación legal requiramos seguir tratando sus datos personales. Asimismo, usted deberá considerar qué para ciertos fines, la revocación de su consentimiento implicará que no le podamos seguir prestando el servicio que nos solicitó, o la conclusión de su relación con nosotros.

Para conocer el procedimiento y requisitos para la revocación de su consentimiento, usted podrá ponerse en contacto con nuestro Departamento de Privacidad a través de los medios especificados en el presente aviso.

¿Cómo puede limitar el uso o divulgación de su información personal?

Si usted desea limitar el uso o divulgación de su información personal podrá solicitarlo a nuestro Departamento de Privacidad a través de los medios especificados en el presente aviso.

Adicionalmente, podemos poner a su disposición procedimientos y mecanismos específicos mediante los cuales puede limitar el uso de su información personal. Estos procedimientos y mecanismos específicos se informarán a través de los medios que utilicemos para comunicarnos con usted u otros que consideremos adecuados.

¿Cómo puede conocer los cambios a este Aviso de Privacidad?

El presente Aviso de Privacidad puede sufrir modificaciones, cambios o actualizaciones derivadas de nuevos requerimientos legales; de nuestras propias necesidades por los productos o servicios que ofrecemos; de nuestras prácticas de privacidad; de cambios en nuestro modelo de negocio, o por otras causas.

Nos comprometemos a mantenerlo informado sobre los cambios que pueda sufrir el presente Aviso de Privacidad a través de los medios de contacto que nos proporcione en su registro.

Consiento que mis datos personales sean tratados de conformidad con los términos y condiciones informados en el presente Aviso de Privacidad.

Nombre y firma del titular:

____
